# Association of myelinated primary afferents impairment with mechanical allodynia in diabetic peripheral neuropathy: an experimental study in rats

**DOI:** 10.18632/oncotarget.19359

**Published:** 2017-07-18

**Authors:** Chenlong Liao, Min Yang, Wenxiang Zhong, Pengfei Liu, Wenchuan Zhang

**Affiliations:** ^1^ Department of Neurosurgery, Xinhua Hospital Affiliated to Shanghai JiaoTong University School of Medicine, Shanghai, P. R. China

**Keywords:** diabetic peripheral neuropathy, nerve decompression, mechanical allodynia, neuropathic pain

## Abstract

To investigate the mechanisms underlying the efficacy of surgical treatment for painful diabetic peripheral neuropathy. Rats were initially divided into 3 groups (I, control rats, II, streptozotocin-induced diabetic rats, III, streptozotocin-induced diabetic rats with latex tube encircling the sciatic nerve without compression). When mechanical allodynia (MA) became stable in the third week, one third of group III rats were sacrificed and the remainder were further divided into subgroups depending on whether the latex tube was removed. Except for some rats in group III, all rats were sacrificed in the fifth week. Morphometric analysis of nerve fibers was performed. Expression level of GABA_B_ receptor protein in spinal dorsal horn was determined. Changes of GABA_B_ receptor within areas of primary afferents central terminal were identified. Chronic nerve compression caused by the interaction of diabetic nerves swelling and the encircling latex tube increased the incidence of MA in diabetic rats, and nerve decompression could ameliorate MA. In diabetic rats with MA, demyelination of myelinated fibers was noted and reduction of GABA_B_ receptor was mainly detected in the area of myelinated afferent central terminals. MA in DPN should be partially attributed to compression impairment of myelinated afferents, supporting the rationale for surgical decompression.

## INTRODUCTION

As a hallmark of neuropathic pain, mechanical allodynia (MA) is not uncommon in patients with painful diabetic peripheral neuropathy (DPN) [1], posing multidisciplinary therapeutic challenges. The outcome of pharmacological treatment is far from satisfactory due to limited efficacy and high rate of side effects [2]. As an alternate treatment, surgical decompression of multiple peripheral nerves [3] based on the “double crush” hypothesis [4] was proposed and has proven to be effective in relieving pain [5, 6]. It is well accepted that diabetic neuropathic pain is caused by peripheral neuropathy [7] and a series of peripheral mechanisms were reported to underlie the development of painful DPN. Central changes, on the other hand, have also been considered as potential mechanisms [2, 8, 9]. Therefore, the effect of surgical decompression of peripheral nerves has been challenged and the mechanisms underlying the efficacy of surgical treatment for painful DPN remained uncertain and controversial. Ideally, both peripheral (input) and central (transmission) mechanisms should be considered together to provide a comprehensive understanding of underlying pathological processes and to develop better therapeutic strategies.

MA is well accepted to be mediated largely by myelinated Aβ-fibers [10]. Abnormal Aβ sensory input is reported to play an important role in diabetic neuropathic pain [7]. According to the gate control theory of pain, [11, 12] input of the myelinated Aβ-fibers is gated by feed-forward activation of inhibitory neurons [11, 12] Hence, as suggested by central mechanisms, MA might be attributed to imbalance between excitatory (mostly glutamate) and inhibitory (mostly GABA and glycine) neurotransmitters in the spinal cord [8, 13].

The GABA_B_ receptor is widely distributed in the central nervous system including the spinal cord [14], and serves as autoreceptor for feedback regulation of synaptic GABA release as well as heteroreceptors that regulate synaptic glycine and glutamate release to the spinal dorsal horn neurons [15–17]. It is hypothesized that downregulation of presynaptic GABA_B_ receptor at afferent terminal is involved in diabetic neuropathic pain by increasing glutamatergic input to the spinal dorsal horn neurons [18].

In the light of previous clinical and basic research, the present study was conducted with the intention to investigate the mechanisms accounting for the efficacy of surgical decompression of peripheral nerves for painful DPN.

## RESULTS

### Rat phenotype after streptozotocin injection

**Figure 1:**
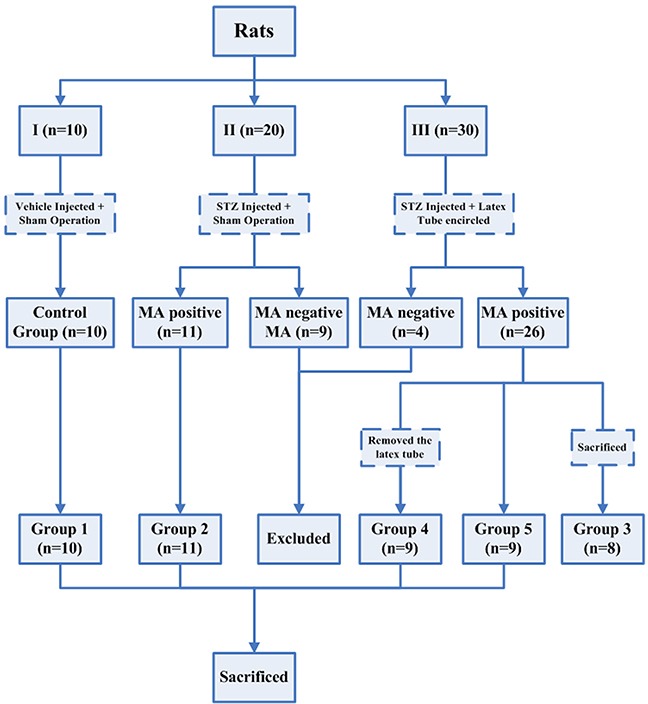
Flowsheet showing different groups of rats that were subjected to different treatment throughout the experimental period

The rats (n=3) that died or failed to develop hyperglycemia following streptozotocin (STZ) injection were excluded from this study (not shown in Figure 1). 10 rats in group I served as control group (group 1). 20 rats in group II and 30 rats in group III developed hyperglycemia (blood glucose >350mg/dL) and displayed polyuria, a reduced growth rate and a marked increase in food and water intake throughout the experimental period, but otherwise the rats remained relatively healthy. Different degrees of nerve swelling, which render the diabetic nerve susceptible to chronic compression by the latex tube, could be noted in all diabetic rats during the nerve harvest procedures at the time of sacrifice. During the surgical procedure, the site of ischial notch where the sciatic nerve passes through was identified as a potential compression site for diabetic swelling nerve since it is quite narrow in nature.

### Mechanical compression increased the incidence of mechanical allodynia in diabetic rats

**Figure 2:**
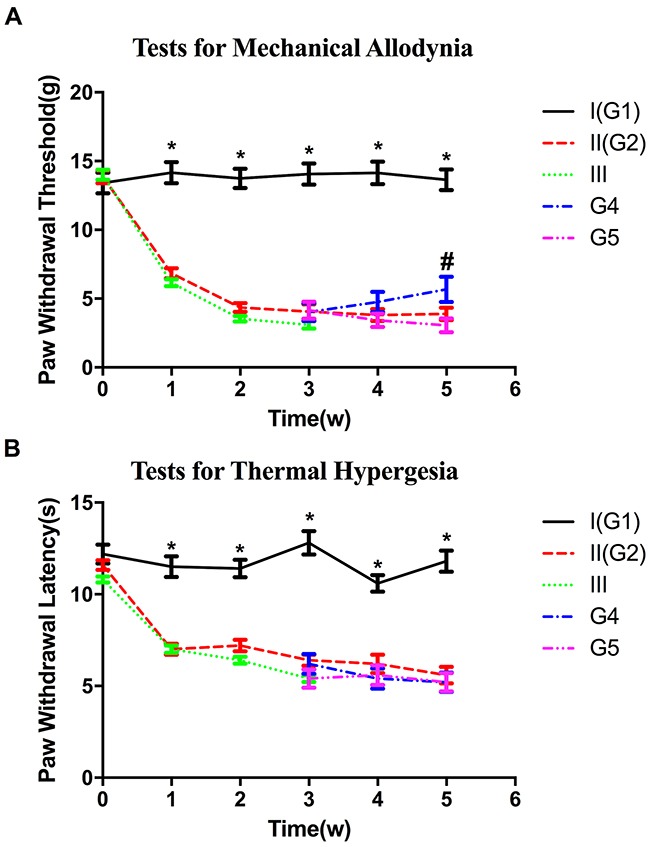
Diagram of behavioral tests showing the changes in the paw withdrawal thresholds **(A)** and paw withdrawal latency **(B)** in all groups. *P<0.05 compared with respective experimental groups values. # *P*<0.05, group 4 versus group 5.

The results of behavioral tests were shown in Figure 2. After 3 weeks, 11 rats in group II, without latex tube enclosure, and 26 rats in group III developed persistent MA. Eight rats in group III, with latex tube encirclement, were sacrificed in the third week (group 3) while the other 18 group III rats were further randomly and evenly divided into two groups, becoming Groups 4 and 5, which had latex tube removal or retention. More diabetic rats with latex tube surrounding the sciatic nerve developed MA (26/30) than those with sham operation (11/20, *P*=0.012<0.05).

The average paw withdrawal threshold (PWT), measured with von Frey filaments, were significantly lower in both group II (4.35±0.19) and group III (4.34±0.13) two weeks after STZ injection when compared with that in control group (13.743±0.43, *P*<0.05, *P*<0.05). In the third week, no significant difference on PWT was noted between group II and III (*P*>0.05). Meanwhile, as regard to the subgroup 4 and 5, no significant difference on PWT was noted (*P*>0.05). By the fifth week, recovery of higher PWT was observed in group 4 with tube compression removal when compared with that in group 5 (*P*<0.05) (Figure 2A).

### Mechanical compression did not increase the incidence of thermal hyperalgesia in diabetic rats

Most rats in group II (19/20) and III (28/30) developed persistent thermal hyperalgesia at 3 weeks after induction of diabetes. The paw withdrawal latency (PWL) of control group is higher than any experimental group (*P*<0.05). No significant difference in PWL was noted among the experimental groups 2,3,4 and 5 during the experimental period (*P*>0.05) (Figure 2B).

### Varying degrees of demyelination of myelinated A-fibers was observed in diabetic rats with mechanical allodynia

**Figure 3:**
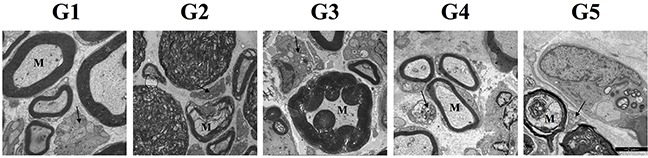
Electron micrographs of sciatic nerves in all groups The black arrows in micrographs point to the unmyelinated fibers and the letter “M” indicates myelinated fibers. Demyelination and degeneration of myelinated fibers and unmyelinated fibers could be noted in the 4 experimental groups. Abnormal morphological structures of unmyelinated fibers, manifesting as dense and collapse configuration, and different degree of myelin impairment could be noted in the experimental groups.

Compared with the control group, an increased degree of demyelination of myelinated fibers was observed in all the experimental groups (Figure 3). As summarized in Table 1, more reduction in myelinated fiber area and density were noted in all the experimental groups when compared with the control group(*P*<0.05). Both myelinated fiber area and density in group 3 were larger than those in group 5 with more prolonged compression (3rd week vs 5th week). Myelinated fiber area and density were larger in group 4 than those in group 2 and 5 (*P*<0.05). Correspondingly, higher g-ratio (axon area/fiber area), which is an indication of demyelination, was noted in all the experimental groups when compared with the control group (*P*<0.05). G-ratio was higher in group 3 than that in group 5. When compared with group 2 and 5, higher g-ratio was noted in group 4. On the other side, degeneration of unmyelinated axons, demonstrated by the morphological changes, could be noted in all the experimental groups. While higher unmyelinated axons area and density was observed in the control group than all experimental groups(*P*<0.05), no significant difference in unmyelinated axon area and density were noted among the four experimental groups (*P*>0.05).

**Table 1:** Morphometric data of both myelinated and unmyelinated fibers in all groups

Groups	MF density (number/mm^2^)	MF axon area (μm^2^)	MF fiber area (μm^2^)	MF g-ratio	UA area (μm)	UA density (number/mm^2^)
1	7863 ± 162^#^	26.83 ± 2.74	67.4 ± 3.4^#^	0.54 ± 0.09^#^	0.76 ± 0.04^#^	8,2637 ± 5372^#^
2	4045 ± 157	25.26 ± 2.33	42.8 ± 2.7	0.66 ± 0.15	0.46 ± 0.07	5,7382 ± 4726
3	4828 ± 168*	25.65 ± 2.16	45.2 ± 2.5*	0.68 ± 0.14*	0.47 ± 0.04	6,0328 ± 4636
4	4652 ± 188^†‡^	26.42 ± 2.08	49.3 ± 2.3^†‡^	0.60 ± 0.11^†‡^	0.44 ± 0.03	5,3804 ± 5022
5	3870 ± 211	24.68 ± 2.24	39.6 ± 2.1	0.75 ± 0.23	0.42 ± 0.04	5,0173 ± 4883

Data are expressed as mean ± SEM. MF, myelinated fiber; UA, unmyelinated axon.

^#^*P*<0.05 Group 1 versus group 2-5;*P<0.05 Group 3 versus group 5; ^†^*P*<0.05 Group 4 versus group 5; ^‡^ *P*<0.05 Group 4 versus group

### Downregulation of GABA_B_ receptor was found in all the diabetic rats with mechanical allodynia and was mainly detected in the area of myelinated afferent terminals

**Figure 4:**
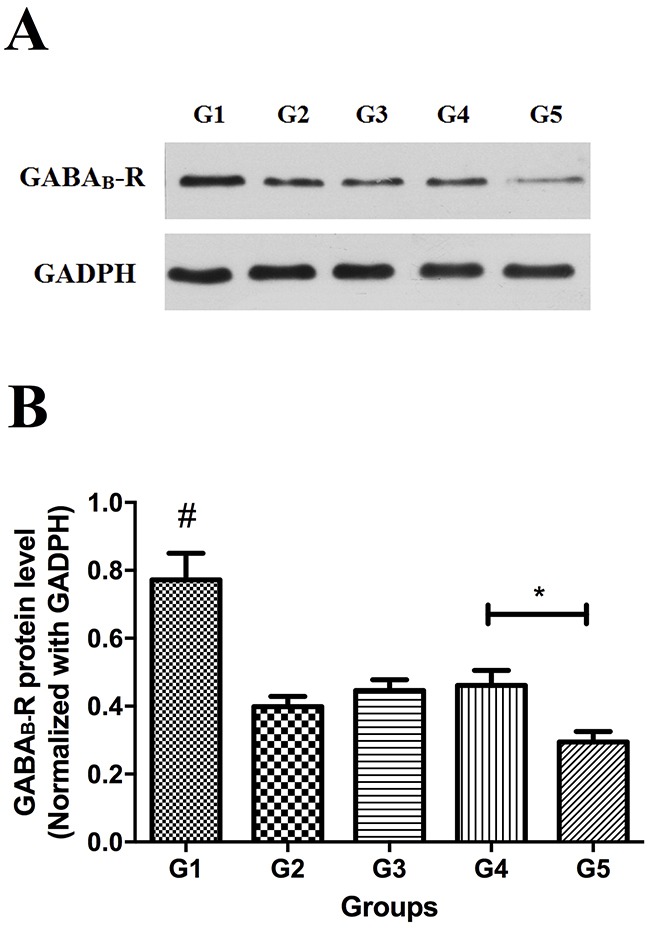
Western blot analysis of GABA_B_ receptor protein in spinal cord (L5) in all groups **(A)** Representative bands, **(B)** summary data. * *P*<0.05, group 4 versus group 5. #, control group versus experimental groups.

Lower expression level of GABA_B_ receptor protein was found in all experimental groups compared to the control group (*P*<0.05). Although it remained at a low level, expression of GABA_B_ receptor protein in group 4 was higher than that in group 5 (*P*<0.05) (Figure 4).

**Figure 5:**
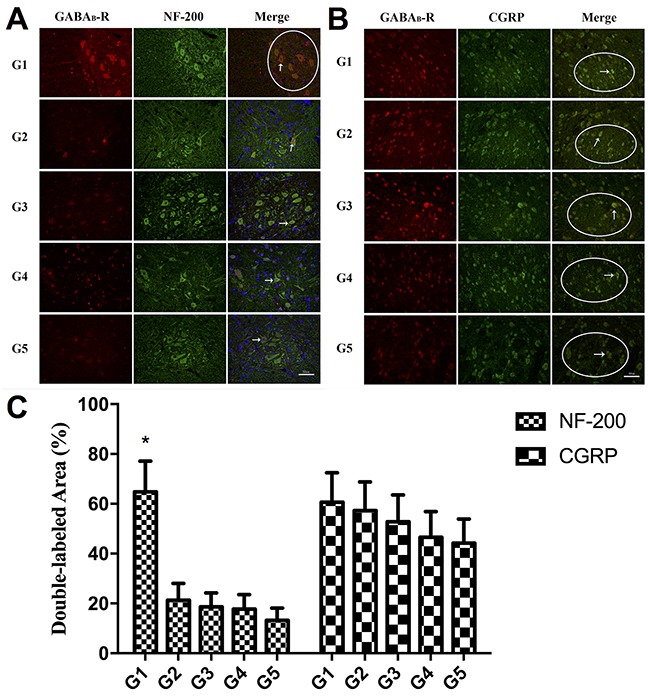
**(A)** Double immunofluorescence staining of GABA_B_ receptor protein and NF-200 in the spinal dorsal horn (L5). Fluorescence of GABA_B_ receptor in the areas of NF-200^+^ neurons can be observed in all the groups (white arrow). In contrast to the control group, in which fluorescence of GABA_B_ receptor can be noted in the areas of NF-200^+^ myelinated afferent terminals (white circle area), little fluorescence staining of GABA_B_ receptor can be seen in the areas of NF-200^+^ myelinated afferent terminals in the 4 experimental groups. Scale bar: 50um. **(B)** Double immunofluorescence staining of GABA_B_ receptor protein and CGRP in the spinal dorsal horn (L5). Immunofluorescence staining of GABA_B_ receptor could be observed in both areas of CGRP^+^ neurons (white arrow) and CGRP^+^ unmyelinated afferent terminals (white circle area) in all groups. Scale bar: 50um. **(C)** The histogram showing the area percentages of double-labeled primary afferents terminals by GABA_B_ Receptor and NF-200 or CGRP in all the GABA_B_ Receptor labeled primary afferents terminals. * *P*<0.05, control group versus group 2-5.

Figure 5 shows the immunofluorescence out-come in the spinal dorsal horn of all groups. While fluorescence of GABA_B_ receptor in the areas of NF-200^+^ neurons, albeit faint, could still be observed in the 4 experimental groups, it could hardly be noted in the areas of NF-200^+^ *myelinated* afferent terminals (Figure 5A). On the other hand, immunofluorescence staining of GABA_B_ receptor could be observed in both areas of CGRP^+^ neurons and CGRP^+^ *unmyelinated* afferent terminals in all groups (Figure 5B). Figure 5C demonstrated the area percentages of double-labeled central terminals of primary afferents by GABA_B_ receptor and NF-200 or CGRP in all the area of GABA_B_ receptor labeled primary afferents terminals. The area of double-labeled primary afferents terminals by GABA_B_ Receptor and NF-200 was larger in the control group compared to the other 4 experimental groups (*P*<0.05). On the contrary, no significant difference in double-labeled primary afferent terminal area was noted among all the experimental groups.

## DISCUSSION

Since peripheral nerves in diabetes are proven to be susceptible to mechanical compression [19], a modified rat model of chronic nerve compression, in which the diabetic sciatic nerve distal to the ischial notch was surrounded by a 1.5-cm length of latex tube [20, 21], was used for this study. Nerve trunk swelling as diabetes is induced exposes the rat nerve to progressive chronic compression by the encircling latex tube, as occurs in human diabetes at known sites of fibro-osseous anatomic narrowings, such as carpal, cubital and tarsal tunnels [22].

In the present study we have found that chronic nerve compression increases the incidence of MA in diabetic rats (group II V.S. III) and in turn, nerve decompression after 2 weeks of hyperglycemia decreases MA as measured by PWT (group 4 vs 5). These data verified that MA in DPN does indeed result, at least partly, from nerve compression. Other salient findings are that demyelination of myelinated fibers was observed in diabetic rats with MA, and that downregulation of GABA_B_ receptor, reported previously to be involved in the development of MA in DPN [18], was mainly detected in the area of myelinated afferent terminals in spinal cord dorsal horn. Combining the morphological impairments in peripheral nerve fibers with the molecular changes in central terminals, we hereby suggest that MA in DPN is associated with structural and functional alteration of myelinated afferent fibers. Consistent with the prior understanding that large myelinated A-fibers are more sensitive to mechanical compression than small unmyelinated C fibers [23, 24], these findings of the present study strongly imply that myelinated A-afferent fibers, rather than unmyelinated C-afferent fibers, are the axons impaired by chronic nerve compression in DPN and lead to the development of MA. Accordingly, the rationale of surgical decompression for painful DPN is vindicated and the reported benefit explained.

Confirming previous studies [25], this report demonstrates that demyelination and degeneration of nerve fibers are prominent features in DPN, resulting in increased g-ratio of axon area/fiber area, and reduction in myelinated fiber area, and density of both myelinated and unmyelinated fibers. Myelinated fiber impairment, we attributed largely to the compression since decompression of the nerve would delay and/or restore the impairment, as shown by larger myelinated fiber area and density in group 4 than in group 5. During the surgical procedure, we identified the site of ischial notch as one of the potential anatomic compression sites in rats of group 2. A swelling sciatic nerve could be compressed here in the setting of diabetes, causing pain symptom. This may also explain why would diabetic rats without latex tube surrounding the sciatic nerve still develop painful DPN. In this way, the result that not all the rats develop painful DPN may owe to the anatomic distinction that not all the diabetic nerve would be compressed by the ischial anatomic narrowing. It might be similar in clinical practice.

Both myelinated fiber area and density in group 3 by the 3rd week sacrifice were larger than those in group 5 at 5 weeks, so impairment of myelinated fibers in painful diabetic rats appears time-dependent. Therefore, patients with painful DPN would benefit most from surgical decompression in an early stage. Early surgery is also supported by several previous clinical reports [26–28].

In addition to MA a, thermal hyperalgesia was likewise induced in most of the diabetic rats. Morphological changes of unmyelinated C-fibers, the main thermal nociceptor [29], could be observed in all groups (Figure 3). However, these changes might not be attributed to mechanical compression since unmyelinated C-fibers are more vulnerable to inflammatory injury [24], which is common in diabetes [30]. This interpretation is supported by our finding that mechanical compression did not increase the incidence of thermal hyperalgesia in diabetic rats, and that decompression ameliorated the degree of demyelination of myelinated axons but not the morphological changes of unmyelinated axons.

In the present study we investigated both peripheral (primary afferent fibers) and central (spinal dorsal horn) changes of nervous systems in rat DPN. Our results suggest that it is the myelinated A-afferent, rather than unmyelinated C-fibers, which are involved in the development of MA in DPN. This is consistent with the results from the Kahn et al functional and structural study on the important role of myelinated afferent nerves in allodynia caused by diabetic neuropathy [7]. Although MA represents a cardinal feature of neuropathic pain, it is only one variety of pain symptom. Patients with DPN describe several other pain types such as aching, burning, painful tingling, lancinating pains and hyperalgesia that can seriously reduce their quality of life [31]. While spontaneous pain has been variously described as burning, shooting, lancinating or crawling in character [2], the evoked pains, including allodynia and hyperalgesia, are often further distinguished according to the different sensory modalities (touch, pressure, pinprick, vibration, cold, heat) that are used to elicit them [32].

Also, it is still a clinical conundrum that why some patients with DPN experience distressing pain while others develop the insensate symptom numbness [33]. Although the natural course of painful diabetic neuropathy is variable [34] and neuropathic pain could be present at any stage of DPN [35], it was suggested that positive symptoms, such as pain and hyperalgesia, is generally present in early stage of DPN, whereas advanced stage is typically characterized by insensate symptoms, such as mechanical and thermal hypoalgesia [36–38]. This time-dependent behavioral switch should be attributed to the consequence of different degree of nerve injury that may range from active degeneration or impaired regeneration to loss of sensory fibers [39]. Although multiple metabolic mechanisms were reported to participated in this process [40–45], demyelination caused by mechanical compression shown in the present study should not be ignored. The evidence comes from the comparison between models of STZ-diabetic rats and mice. STZ-diabetic rats were reported to develop thermal and mechanical hyperalgesia within 2-8 weeks and become hypoalgesic after longer periods of diabetes [40, 46, 47] while hyposensitivity to heat and mechanical stimuli, rather than hyperalgesia and MA, was found in STZ-treated mice [48–50]. Correspondingly, in early stage, signs of demyelination could be observed in STZ-diabetic rats as shown in the present study and other previous reports [51, 52], but not in diabetic mice [53]. Nevertheless, neuropathic pain can be induced in several nerve compression models in mice, such as chronic constriction injury model, spinal nerve ligation model and spared nerve injury model [54–56], suggesting that diabetic nerve in rats is compromised by both metabolic inflammation and mechanical compression while that in diabetic mice may be impaired by solely metabolic inflammation. This species-dependent difference on positive and negative symptoms may result from either distinction of anatomic narrowings or the different susceptibility of the nerves to compression in the setting of DPN.

The diverse characteristics of pain symptoms could imply that a number of mechanisms are involved, with various patients (even in each stage of DPN) having a differing pathogenic profile. However, mechanisms of neuropathic pain are neither disease-specific nor symptomatic-specific [57, 58]. As classified by Spallone et al., in the case of peripheral neuropathic pain, damage to peripheral sensory nerves and subsequent primary afferent activity was considered as the initial event, with the manifestations of pain symptoms resulting from consequent changes in structure and function of the somatosensory nervous system [59]. This may be the reason why large numbers of previous studies failed to identify a significant functional or pathological abnormality related to pain in diabetic neuropathy [60, 61].

Making a distinction between spontaneous pain and evoked pain is difficult in both clinic work and laboratory research. According to the Jensen and Finnerup, “it would be impossible to establish whether a stimulus is capable of activating nociceptors in the individual patient” [32] and in turn, patients may take provoked pain as spontaneous pain in daily life and thus provide inaccurate information about the characteristic of pain. In parallel, it is not possible to quantify spontaneous pain in animals [33] and reduced PWT or PWL is frequently presumed as a general pain symptom in many models of pain. Therefore, while we have demonstrated surgical decompression is effective in ameliorating MA, we also show that decompression is not effective for all types of DPN pain. It seems appropriate to view DPN as a heterogenous disease state, and other mechanism-based multimodal treatments should be further investigated.

Sciatic nerve compression in streptozotocin-induced diabetic rats produces mechanical allodynia and thermal hyperalgesia manifest in paw withdrawal. These objective behavioral changes correlate with sciatic nerve axonal Aβ-fiber demyelination and degeneration of unmyelinated axons. Downregulation of GABAB receptor was present in all diabetic rats with mechanical allodynia in the spinal cord areas of myelinated afferent terminals. Surgical decompression by removal of a sciatic encircling latex tube reduced allodynia but not thermal hyperalgesia.

A role of compression impairment of myelinated afferents in producing allodynia and the benefit of decompression is confirmed, revealing the rationale of surgical decompression. This demonstrated amelioration of allodynia provides justification for clinical use of external neurolysis of fibro-osseous tunnel compression sites in painful human diabetic peripheral neuropathy.

## MATERIALS AND METHODS

### Study approval

All procedures involving animals were in accordance with the guideline for the Care and Use of Laboratory Animals. This study protocol was approved by Ethics Committee of Xinhua Hospital (NO. XHEC-F-2016-009). All efforts were made to minimize both the suffering and number of animals.

### Animals and experimental design

Male Sprague-Dawley rats initially weighing 150-180 g were used. The rats were housed at a temperature of 21-24°C and maintained a 12-h light/dark cycle throughout the experimental period. The rats were initially randomly divided into 3 groups according to the different treatments (Figure 1). Diabetes was induced in rats of groups II and III by a single intraperitoneal administration of STZ (60mg/kg; Sigma, St. Louis, MO, USA) freshly dissolve in 0.1 mol/L citrate buffer (pH 4.5). Weight-matched vehicle (sodium citrate buffer) was injected into the rats of control group. Diabetes was confirmed three days later by measurement of plasma glucose concentration (>350mg/dL) in blood samples obtained from the tail vein. The glucose level was assayed enzymatically using Sigma diagnostic glucose reagents.

To test the hypothesis that the underlying metabolic abnormalities involved in diabetes render the peripheral nerve susceptible to chronic nerve compression, a modified rat model of chronic nerve compression was used in this study to investigate the effect of mechanical compression on the development of MA caused by DPN [20] All surgical procedures were performed under aseptic conditions by one surgeon (Liao). The rats were anesthetized with an intraperitoneal injection of sodium pentobarbital (50mg/kg), followed by the induction of diabetes. The hair in the right thigh region was removed and the skin was sterilized with 0.5% povidone solution. The skin and muscle layer of the lateral surface of the right thigh was incised and the sciatic nerve was exposed. A 1.5cm-long latex tube was used to surround the sciatic nerve on the distal site of ischial notch. The tube was sutured with three sutures of 7-0 nylon, thereby encircling without compressing the nerve (the tube could be easily moved back and forth along the nerve). Muscle and skin layer was sutured with silk thread and topical antibiotic powder was applied at once.

This model of chronic nerve compression was built in rats of group III while sham operation was performed in rats of group I and II. The diabetic rats in group II and group III that did not develop MA within 3 weeks were excluded. Meanwhile, one-third rats with MA in group III were sacrificed (as group 3) at the end of the third week and the remainder were then further divided into two groups (as group 4 and 5). After 3 weeks, latex tube was removed in rats of group 4 but was retained in rats of group 5. Excluding the group 3 rats sacrificed in the third week, all other rats were sacrificed in the fifth week (Figure 1). The sample size and time points were based on the results of our preliminary experiments and the power analysis.

### Behavioral analysis

MA and thermal hyperalgesia were tested weekly by two observers blinded to the experimental groups. The observers were not blind to control group and experimental groups due to the distinct physical appearance of diabetes.

MA was determined by quantifying the PWT in response to von Frey filaments (Stoelting, Wood Dale, USA). The rats were acclimated for 30 minutes in suspended individual chambers on a mesh floor. A series of calibrated von Frey filaments, starting from the lowest force filament, were applied perpendicularly to the plantar surface of the hindpaw for 6 seconds with sufficient force to bend the filament. Brisk withdrawal or paw licking was considered as a positive response. In the absence of a response, the filament of next greater force was applied. In the presence of a response, the filament of next lower force was applied. The mechanical stimulus producing a 50% likelihood of withdrawal response was calculated by using the ‘up-and-down’ method as described previously [62, 63]. The lowest force filament able to produce a positive reaction is determined based on 3 positive responses in at least 5 repeated measurements. Each testing was performed at 2-min intervals to determine a mean value.

Thermal hyperalgesia was determined by PWL in response to radiant heat. Rats were placed within plexiglass enclosures on a transparent 30°C glass surface. After acclimatizing for 30 minutes, a thermal testing apparatus (IITC/Life Science, woodland Hills, CA, USA) was used to apply radiant noxious heat to the plantar surface of the hindpaw. The latency time to paw withdrawal was measured by a digital timer. Testing was repeated 3 times at 2-min intervals to determine a mean value for the thermal threshold. A no-response cut-off of 30 seconds was used to prevent potential tissue damage [64].

### Morphological study

Before sacrifice, rats were anesthetized as described above and the right sciatic nerves distal to the ischial notch were quickly transected and separated into two segments. One was post-fixed overnight in 4% paraformaldehyde and dehydrated stepwise in increasing concentration of ethanol prior to embedding in paraffin. A 1.5μm transverse section was cut on a Leica RM2016 microtome, stained with toluidine blue and examined under a light microscope. The second was fixed in 2.5% glutaraldehyde solution. After 24 hours, the tissues were transferred into phosphate-buffered saline (pH 7.4) and then were post-fixed in 1% osmium tetroxide for 2 hours, dehydrated stepwise in increasing concentration of ethanol followed by propylene oxide. The tissues were finally impregnated with a series of mixtures of resin and propylene oxide and embedded in 100% epoxy resin. The semithin sections (<1μm) were examined under an electron microscope.

The number and density of myelinated fibers was counted on the light microscope montages. Morphometric assessments were made on about 500 myelinated fibers in each specimen. Electron micrographs (×3000) of at least 300 myelinated fibers were prepared for each specimen to calculated the myelinated axon and fiber area and g-ratio (axon area/fiber area). Electron micrographs (×10, 000) of at least 30 unmyelinated fibers were collected by a systematic random sampling technique [61]. The unmyelinated axons were identified with the use of established criteria, including the presence of neurotubules and neurofilaments defined from electron micrographs at high magnification and the circularity and distribution of the axons enveloped by the Schwann cell [65]. The number and diameter of unmyelinated axons were directly counted and the density was derived from the total endoneurial area assessed. Calculation was performed with the use of AnalySIS Pro 3.1 (Soft Imaging Systems GmbH, Munster, Germany) by two observers who were blinded to the groups. All rats in each group were used in morphological assessments (Figure 1).

### Western blot

The L5 spinal cord was transected on ice and the tissue was homogenized in protease inhibitor cocktail (Sigma, St. Louis, MO) and RIPA lysis buffer. An equal amount of protein (60 μg) was loaded into each lane, separated electrophoretically by SDS-polyacrylamide gel and transferred to polyvinylidene fluoride membranes. After washing in TBS with 0.1% Tween-20 (TBS-tween), the membranes were blocked with 5% nonfat milk dissolved in TBS-tween for 1 hour at room temperature and incubated overnight at 4°C with rabbit monoclonal anti- GABA_B_ Receptor 1 antibody (1:500, Abcam, catalog NO. ab55051) and anti-GADPH (1:1000, Wuhan goodbio technology, Wuhan, China). After several washes in TBS-tween, the membranes were incubated with horseradish peroxidase-conjugated goat anti-rabbit secondary antibody (1:3000, Wuhan goodbio technology, Wuhan, China) for 1 hour at room temperature. Finally the membranes were washed in TBS-tween and chemiluminescent bands were detected using ECL technology (Wuhan goodbio technology, Wuhan, China; G2014). The intensity of the bands was captured digitally and analyzed quantitatively by a blinded observer using the alphaEaseFC (Alpha Innotech) and Adobe PhotoShop (Adobe). All GABA_B_ receptor protein concentrations were normalized to GADPH protein. Specimens from half number of the animals in each group were used in western blot.

### Immunofluorescence

Under deep anesthesia, rats were then transcardially perfused with 150ml of saline followed by 250ml of 4% paraformaldehyde in 0.1M phosphate-buffered saline (pH 7.4). The L5 spinal cord was quickly transected and postfixed by immersion in the same fixative solution overnight. The spinal cord tissue was dehydrated, embedded in paraffin and sectioned at a thickness of 5 μm. The sections were preincubated in blocking solution (10% goat serum, 1% Triton X-100 in PBS) for 30 minutes at room temperature, followed by overnight incubation at 4°C with rabbit monoclonal anti-GABA_B_ Receptor 1 antibody (1:400, Abcam, catalog NO. ab55051) and either mouse monoclonal anti-Hypophosphorylated neurofilament H antibody (anti-NF-200, a marker for myelinated A-fibers [66], 1:200, Abcam, catalog NO. ab82259) or mouse monoclonal anti-Calcitonin gene related peptide antibody (anti-CGRP, a marker for unmyelinated C-fibers [67], 1:100, Abcam, catalog NO. ab81887) in blocking solution. After three washes with PBS, the fluorophore-conjugated secondary antibodies, including Alexa Fluor 647 AffiniPure Goat Anti-Rabbit IgG antibody (1:100, Jackson) and FITC-AffiniPure Goat Anti-mouse IgG antibody (1:50, Jackson) were applied for 1 hours at room temperature. Cell nuclei were counterstained with DAPI. Control experiments determining the specificity of the immunofluorescence were performed by omitting the primary antibodies. The stained sections were examined with the Olympus FluoView 1000 confocal laser scanning microscope. In spinal dorsal horn, the areas of central terminals of primary afferents that were double labeled with GABA_B_ Receptor and NF-200 or CGRP were calculated to detect the changes of presynaptic GABA_B_ receptor level within the central terminals of two different primary afferents with the use of Image Pro plus 6.0 (Media Cybernetics, Silverspring, USA). Specimens from half number of the animals in each group were used in immunofluorescence assay, which was performed on 5 sections from each animal. Observation was performed within the area of laminae I-V and at least 5 areas of each section were evaluated.

### Statistical analysis

Comparison between the number of rats developing MA and thermal hyperalgesia in different groups were analyzed with the Pearson's χ^2^ test. The quantification of protein expression, fluorescence staining and the morphometric analyses were performed with the use of the one-way ANOVA followed by Tukey's post-hoc test. Repeated measurement Two-way ANOVA with Tukey's post-hoc test was used to determine the levels of blood glucose, changes of PWT and PWL in different groups along the experimental period. All the data were expressed as mean ± SEM. Statistical analyses were completed with SPSS 18.0. The criterion for statistical significance was *P*<0.05.

## Abbreviations

DPN: diabetic peripheral neuropathy
MA: mechanical allodynia
PWL: paw withdrawal latency
PWT: paw withdrawal threshold
STZ: streptozotocin
